# Trends and Outcomes in Technology-Assisted Total Knee Arthroplasty in the United States From 2020 to 2024

**DOI:** 10.1016/j.artd.2026.102064

**Published:** 2026-06-19

**Authors:** Michael S. Kim, Madison Brunette, Emily Pham, Kylen Soriano, Ryan DiGiovanni, Peter Hsiue

**Affiliations:** Department of Orthopaedic Surgery, University of California, Irvine, School of Medicine, Orange, CA

**Keywords:** Total knee arthroplasty, Technology-assisted surgery, Utilization trends, Surgical outcomes

## Abstract

**Background:**

Technology-assisted total knee arthroplasty (TA-TKA), including computer navigation and robotic assistance, has been developed to improve implant alignment and soft tissue balance. While utilization has increased, evidence regarding clinical benefit remains mixed. This study evaluated national and regional utilization trends of TA-TKA and compared postoperative complication rates with manual TKA (M-TKA).

**Methods:**

Patients undergoing primary TKA between January 1, 2020, and December 31, 2024, were identified in the TriNetX United States Collaborative Network. Patient demographics, hospital characteristics, and postoperative complications were obtained. Cohorts were 1:1 propensity score matched for sex, body mass index, and comorbidities. Surgical complications were compared at 0-30 days, 31-90 days, and 91 days-3 years between M-TKA and TA-TKA cohorts. In addition, subanalysis was conducted comparing 3-year surgical complication rates between TA-TKA and M-TKA, stratified by region.

**Results:**

Of 198,263 TKA patients, 147,156 underwent M-TKA and 39,624 underwent TA-TKA. TA-TKA utilization increased by 375%, from 11.9% in 2020 to 27.0% in 2024 (*P* < .0001). After matching, TA-TKA was associated with lower risks of periprosthetic fracture, deep infection, revision surgery, and aseptic loosening (all *P* ≤ .031). Across all regions, statistically significant findings demonstrated decreased risk of surgical complications at 3 years associated with TA-TKA when compared to M-TKA (*P* < .001).

**Conclusions:**

TA-TKA utilization has more than doubled in recent years, though adoption remains variable across regions and institutions. Compared with M-TKA, TA-TKA was associated with significantly lower surgical complication rates through 3 years of follow-up. These findings support continued adoption of TA-TKA and highlight the need for further research on long-term outcomes and cost-effectiveness.

## Introduction

Total knee arthroplasty (TKA) is a widely performed and effective intervention for patients with knee osteoarthritis, with over one million procedures conducted annually in the United States. [1] Despite its success, achieving optimal clinical outcomes remains challenging, with implant positioning and soft tissue balance being critical determinants. [2] Technology-assisted total knee arthroplasty (TA-TKA), which includes computer-assisted navigation and robotic assistance, has emerged as a tool to enhance the accuracy and precision of implant placement and quantify intraoperative balancing. [[3], [4], [5]] Reducing these complications may have the potential to improve outcomes and may be associated with lower revision rates and related morbidity and healthcare costs.

TA-TKA provides real-time intraoperative feedback on alignment and ligament balancing, allowing for more personalized, data-driven surgical decisions. [6] However, the adoption of TA-TKA has been tempered by concerns regarding increased operative time, steep learning curves, and higher associated costs, which may limit widespread use in certain practice settings. [6] Evidence for the clinical benefits of TA-TKA remains mixed; some studies demonstrate short-term improvements in perioperative outcomes, such as improved mechanical alignment, reduced rates of outliers, and more consistent restoration of joint kinematics, while others find no significant difference in long-term survivorship or complication rates when compared to manual techniques. [2,3,[7], [8], [9]] Collectively, these mixed findings and implementation challenges highlight the complexity of assessing the clinical value of TA-TKA and underscore the importance of further research to clarify its impact on surgical outcomes.

Nevertheless, utilization of TA-TKA continues to increase, albeit unevenly across regions. A retrospective national study by Lan et al. reported an increase in TA-TKA rates from 6.2% in 2017 to 8.8% in 2019, with higher adoption in the West and Midwest. [10] However, recent national data remain limited, and prior studies are often constrained by small sample sizes or single-institution designs.

The primary objective of this study was to assess national and regional utilization trends in TA-TKA using a large, national electronic health record database. Secondary objectives included comparing postoperative surgical complication rates between TA-TKA and manual TKA (M-TKA). We hypothesized that TA-TKA utilization has increased over time, albeit with regional and institutional variability, and that TA-TKA is associated with lower rates of surgical complications compared to M-TKA.

## Methods

**Figure 1:**
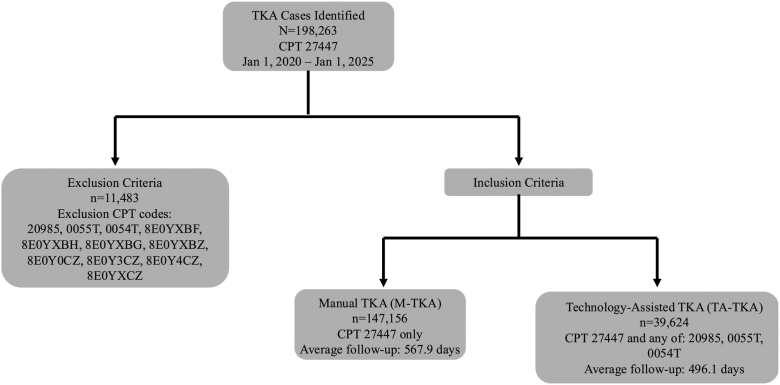
Flowchart depicting cohort identification. CPT, Current Procedural Terminology.

Patients undergoing primary TKA between January 1, 2020, and December 31, 2024, were identified from the TriNetX United States Collaborative Network, a multicenter electronic health record database encompassing over 117 million patients. The M-TKA cohort was identified using Current Procedural Terminology 27447. The TA-TKA cohort was identified using Current Procedural Terminology 20985, 0055T, or 0054T. The average follow-up time was 567.9 days for M-TKA and 496.1 days for TA-TKA (Fig. 1).

Annual procedural incidence, patient demographics, hospital characteristics, and postoperative surgical complication rates were obtained. Hospital characteristics included U.S. region (West, South, Midwest, and Northeast) and institution type (academic vs nonacademic). Patient demographics included age, sex, and race.

Cohorts were 1:1 propensity score matched based on sex, body mass index, and comorbidities (Appendix 1). Postoperative surgical complications were assessed at 0-30 days, 31-90 days, 91 days-3 years, and cumulatively at 0-3 years. Additionally, regional odds ratios (ORs) and 95% confidence intervals for TA-TKA complications were calculated based on aggregate regional frequencies. Comparisons were conducted using 2-tailed Student’s *t*-tests after verifying normal distribution. ORs were calculated, and statistical significance was defined as *P* < .05.

## Results

**Figure 2:**
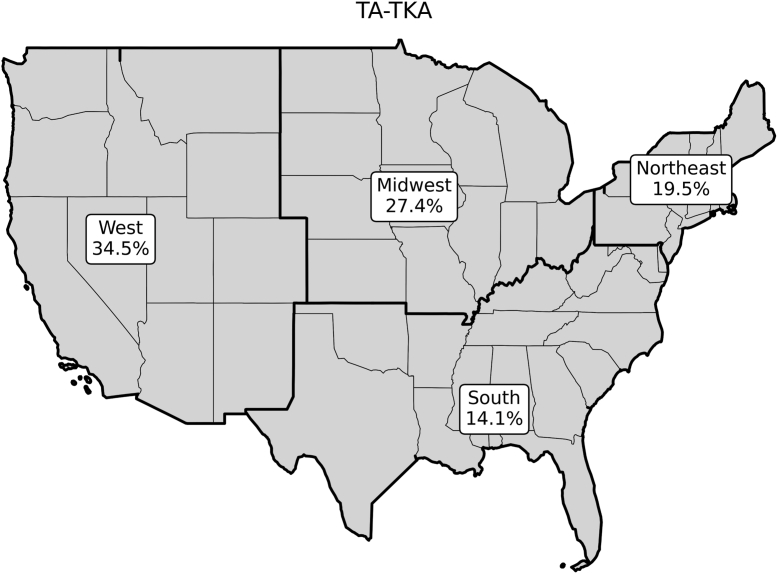
Regional distribution of TA-TKA vs M-TKA utilization in the United States.

Between 2020 and 2024, a total of 147,156 patients underwent M-TKA, while 39,624 underwent TA-TKA. TA-TKA increased by 375%, from 3008 in 2020 to 11,286 in 2024. As a proportion of all TKA, TA-TKA utilization rose from 11.9% in 2020 to 27.0% in 2024). TA-TKA utilization varied significantly by geographic region, with the Western region reporting the highest utilization and the Southern region the lowest (34.5% vs 14.1%, *P* < .0001) (Table 1, Fig. 2). Data from 49 academic and 19 nonacademic healthcare organizations (HCOs) also showed a statistically significant difference in TA-TKA adoption, with academic HCOs reporting higher utilization compared to nonacademic HCOs (21.4% vs 19.7%, *P* < .0001) (Table 1).

**Table 1:** Patient demographics.

	TA-TKA % (N)	M-TKA % (N)
Sex		
Female	53.4 (20816)	58.59 (78145)
Male	35.23 (13734)	37.79 (50408)
Race		
White	70.99 (27673)	72.4 (96569)
Black or African American	5.29 (2062)	11.37 (15161)
Asian	3.68 (1435)	2.55 (3398)
Native American	0.25 (98)	0.24 (323)
Native Hawaiian	0.2 (78)	0.31 (412)
Other	4.16 (1620)	3.46 (4636)
Ethnicity		
Not Hispanic or Latino	72.37 (28212)	72.68 (96936)
Unknown ethnicity	22.69 (8846)	21.75 (29017)
Hispanic or Latino	4.94 (1924)	5.57 (7430)

	Mean Age	Patients
TA-TKA	67.9 ± 9.0	39,003
M-TKA	67.4 ± 9.3	133,516

	BMI	Patients

TA-TKA	31.7 ± 6.02	39,003
M-TKA	32.1 ± 6.11	133,516

BMI, body mass index.

Before propensity score matching, the M-TKA and TA-TKA groups demonstrated notable differences in baseline demographics. The mean age at index surgery was similar between groups (67.5 ± 9.3 years for M-TKA vs 67.9 ± 8.9 years for TA-TKA). A higher proportion of patients undergoing conventional TKA were female (58.6%) compared to the TA-TKA group (53.4%). Racial distribution differed modestly between groups, with a higher percentage of White patients undergoing M-TKA (72.4%) compared to TA-TKA (71.0%). Similarly, Black or African American patients represented a larger proportion of the M-TKA (11.4%) cohort than the TA-TKA cohort (5.3%). No statistically significant differences in patient demographics were identified between the TA-TKA and M-TKA cohorts after propensity score matching.

The TA-TKA cohort exhibited decreased risks of several surgical complications at all postoperative time points. Within 30 days of surgery, patients in the TA-TKA group had a decreased risk of periprosthetic fracture (0.07% vs 0.1%; OR: 0.591; *P* = .031) and deep infection (0.3% vs 0.5%; OR: 0.531; *P* < .001). Between 31 and 90 days, the TA-TKA group had a decreased risk of revision surgery (0.2% vs 0.4%; OR: 0.647; *P* = .001) and deep infection (0.4% vs 0.6%; OR: 0.640; *P* < .001). At the 91-day to 3-year interval, and in the cumulative 0-3 year follow-up period, TA-TKA demonstrated a decreased risk of revision surgery (1.3% vs 1.8%; OR: 0.675; *P* < .001), deep infection (1.0% vs 1.5%; OR: 0.667; *P* < .001), and aseptic loosening (0.3% vs 0.6%; OR: 0.549; *P* < .001) (Table 2). Comparison of 3-year surgical complication rates between TA-TKA and M-TKA stratified by region demonstrated decreased complication rates for TA-TKA. Specifically, there was a decreased risk for revision surgery and deep infection across all regions. There was a decreased risk for superficial infection and aseptic loosening for all regions except for the West where results were not statistically significant. Finally, there was a decreased risk for periprosthetic fracture in the Midwest and South regions; the results for the West and the Northeast regions did not reach statistical significance. Of note, the only increased risk was for periprosthetic fracture in the West, but this did not reach statistical significance (Table 3).

**Table 2:** Regional and institutional utilization of M-TKA vs TA-TKA

	M-TKA % (N)	TA-TKA % (N)
Region		
Northeast	80.5 (56,258)	19.5 (13,610)
Midwest	72.6 (22,384)	27.4 (8462)
South	85.9 (56,663)	14.1 (9290)
West	65.5 (14,512)	34.5 (7638)
Institution		
Academic	78.6 (100,865)	21.4 (27,512)
Nonacademic	80.3 (47,473)	19.7 (11,667)

Chi-squared test result: χ^2^(3) = 524.63, *P* < .0001.

Chi-squared test result: χ^2^(1) = 70.93, *P* < .0001.

**Table 3:** Regional comparison of postoperative complication rates for TA-TKA vs M-TKA.

	West				Northeast				Midwest				South			
	TA-TKA (7638)	M-TKA (14,512)	OR	*P* value	TA-TKA (13,610)	M-TKA (56,258)	OR	*P* value	TA-TKA (8462)	M-TKA (22,384)	OR	*P* value	TA-TKA (9200)	M-TKA (56,663)	OR	*P* value
Knee revision	1.11%	1.70%	0.65	<.001	1.64%	2.31%	0.71	<.001	1.87%	3.51%	0.53	<.001	1.24%	2.13%	0.53	<.001
Periprosthetic fracture	0.37%	0.25%	1.48	.12	0.41%	0.50%	0.82	.15	0.48%	0.78%	0.62	<.001	0.37%	0.54%	0.62	<.001
Deep infection	0.83%	1.60%	0.52	<.001	1.28%	1.62%	0.79	<.001	1.02%	2.78%	0.37	<.001	1.07%	1.91%	0.37	<.001
Superficial infection	0.58%	0.53%	1.09	.63	0.40%	0.57%	0.70	<.001	0.50%	1.10%	0.45	<.001	0.72%	0.71%	0.45	<.001
Aseptic loosening	0.30%	0.34%	0.88	.62	0.26%	0.79%	0.33	<.001	0.57%	0.87%	0.66	<.001	0.47%	0.73%	0.66	<.001

OR compares TA-TKA to M-TKA; OR<1 favors TA-TKA.

## Discussion

This large, national database study demonstrates a substantial and ongoing increase in the utilization of TA-TKA in the United States between 2020 and 2024, with TA-TKA comprising 27.0% of all TKA procedures by the end of the study period. Adoption remains variable, with higher utilization in the Western region and academic institutions, consistent with prior reports of regional and institutional differences in utilization of advanced surgical technologies. The observed lower utilization of TA-TKA among Black or African American patients may reflect disparities in access to technology-assisted procedures, which could be influenced by geographic variation in adoption, institutional resources, insurance coverage, and broader socioeconomic factors; however, these variables were not directly assessed in the present study. These patterns likely also reflect variation in resource availability, surgeon training, and institutional prioritization of innovation. [10] Across all regions, TA-TKA was associated with a decreased risk of most surgical complications compared to M-TKA, although the magnitude of this association varied by region (Table 3). These findings are interesting given that there was variable utilization of TA-TKA by regions. While no causal relationship can be determined, it does suggest that simply utilizing TA-TKA is associated with a decreased risk of surgical complications and that the magnitude of utilization does not matter.

In the acute setting, our analysis identified a statistically significant decrease in early postoperative periprosthetic fracture in TA-TKA compared to M-TKA. Although rare, periprosthetic fractures associated with TA-TKA have been reported. [11] Importantly, current evidence suggests that this association is unlikely to be driven by TA-TKA pin placement. Stetzer et al. demonstrated that intra-incisional pin placement is safe, and LeBrun et al. reported no difference in 90-day complication rates between intra- and extra-incisional pin sites. [12,13] While our study did not specifically control for pin placement, the overall low incidence of fracture suggests that the observed reduction is unlikely to be clinically significant. This aligns with existing literature, which attributes periprosthetic fracture risk more to patient- and surgery-related factors than to robotic pin placement.

Similarly, our analysis proposes that there is a significantly decreased risk of deep infection in the early postoperative period with TA-TKA. While early concerns regarding infection risk in TA-TKA revolved around increased OR time, the current literature does not support this increased risk. [14] Several meta-analyses have reported no significant difference in deep infection within 1 year for TA-TKA compared to M-TKA. [15] Long-term meta-analysis which assessed infection risk with longer term follow (minimum 1 year) also demonstrated no significant difference in both superficial and deep infection between TA-TKA and M-TKA. [16] Given the already low infection rate, it is unlikely that the modest differences observed in our data represent clinically meaningful risk reduction.

Furthermore, our findings demonstrate a decreased risk of revision surgery after TA-TKA, both acutely and at 3 years, compared to M-TKA. In our cohort, the cumulative revision rate was 1.3% for TA-TKA vs 1.8% for M-TKA. Similar reductions have been reported in national registry data. From the American Joint Replacement Registry 2024 Annual report, the cumulative percent revision rate for all cemented TA-TKA and M-TKA at 1 year and 10 years were 0.8% and 2.6%, respectively. [17] Similarly, the Australian Joint Registry reported a 3-year cumulative percent revision rate of 1.8% for TA-TKA which was significantly less than 2.2% for M-TKA. This rate increases to 2.2% and 2.9% at 5 years, respectively. [18] The current published data demonstrate that while the baseline incidence of revision for TKA is already low, there does appear to be a consistently detectable absolute reduction in revision rate in both the acute and long term for TA-TKA. In our cohort, this difference may be partially explained by the lower rates of complications observed with TA-TKA, including deep infection, aseptic loosening, and periprosthetic fracture. However, as revision indications were not directly stratified in this study, the specific cause of revision could not be determined.

While this trend is compelling, analysis demonstrates that the rate reduction may not be clinically significant. Kirchner et al. found that within 2-year follow-up, the odds of revision surgery were unchanged when controlling for confounding variables. [19] As such, it is important to note that surgical outcomes depend on factors beyond specific surgical techniques or technology used. Furthermore, our analysis did not stratify revision indications, limiting our ability to attribute reduced revision solely to improved surgical outcomes.

The current appeal of robotic assistance is through the improved accuracy of bone resection and the ability to intraoperative assessment and adjust planned flexion and extension gaps. [20] Shalhoub et al. demonstrated that TA-TKA consistently achieves mediolateral gap balance within 2 mm of the preoperative plan, and Held et al. showed improved compartment symmetry and less overstuffing compared to M-TKA. [21,22] Cadaveric studies have further confirmed that TA-TKA produces more equivalent medial-lateral load sharing. [23] However, long-term clinical outcomes have not consistently demonstrated superiority of TA-TKA over M-TKA. Together, these advantages of enhanced operative planning and minimized soft tissue trauma may help explain the differences in complication rates observed in our study. These mechanisms, however, were not directly assessed in the present analysis.

Our study also found decreased rates of aseptic loosening with TA-TKA in long-term follow-up, consistent with prior reports. The mechanism underlying this finding is likely multifactorial. As discussed, improved accuracy in implant placement and achieving symmetric compartments likely enhance implant fixation. [21,22] One study by Marchand et al. reported decreased aseptic loosening at 2 years with TA-TKA with use of cementless fixation. [24] Our analysis did not stratify for fixation method (cemented vs cementless), so we cannot attribute our observed decrease in aseptic loosening to fixation method alone. As hypothesized, it is likely a result of the improved implant placement and surgical accuracy that accompanies technology assistance. Longer-term data, including a recent TriNetX study, also support reduced aseptic loosening at 5 years. [25] As such, while our findings are consistent with prior literature, they should be interpreted as an association rather than reflecting a specific underlying mechanism. Further studies evaluating the interaction between technology assistance, fixation method, and long-term implant survivorship are warranted.

Overall, the observed reductions in surgical complications including fracture, revision, and aseptic loosening presented in our study must be assessed carefully. These results should be interpreted within the context of effect sizes and the inherent limitations of large database analyses. While several were statistically significance, the absolute risk reductions were small, reflecting the already low baseline incidence of complications following primary TKA. As such, the clinical significance of these differences remains uncertain.

The present study adds to a growing body of evidence indicating that TA-TKA may improve short- to mid-term outcomes and supports its increasing adoption in clinical practice. However, these findings should be interpreted within the context of several limitations. First, large database studies are inherently limited by coding accuracy, selection bias, and unmeasured confounding variables. While propensity score matching was used to mitigate such biases, residual confounding factors may persist. Additionally, the definition of TA-TKA was based on billing codes, which may not fully capture the nuances of intraoperative factors such as operative time, blood loss, surgical technique, or surgeon experience. Because TA-TKA was identified using billing codes, there is also potential for undercapture if technology assistance codes were not consistently submitted across institutions. Furthermore, the database does not capture surgical alignment philosophy utilized (eg, mechanical vs kinematic alignment), which may differ between TA-TKA and M-TKA and could influence outcomes. Despite these limitations, the strength of this study lies in its large, geographically diverse sample size, robust matching methodology, and longitudinal follow-up. The observed reduction in complications highlights a potential clinical benefit of TA-TKA that warrants further prospective investigation. Future studies should focus on cost-effectiveness, long-term outcomes, and patient-reported satisfaction to further elucidate the value of TA-TKA in routine practice.

## Conclusions

Technology-assisted TKA utilization has markedly increased in recent years and now accounts for over one-quarter of all TKAs performed in the United States. Its adoption varies by region and institution type. This study demonstrated significantly lower rates of surgical complications through 3 years of follow-up. These findings support the continued adoption of TA-TKA and underscore the need for ongoing research to define its long-term clinical and economic impact.

## CRediT authorship contribution statement

**Michael S. Kim:** Writing – review & editing, Writing – original draft, Supervision, Methodology. **Madison Brunette:** Writing – original draft, Visualization, Investigation, Formal analysis, Data curation. **Emily Pham:** Visualization, Investigation, Formal analysis, Data curation. **Kylen Soriano:** Writing – review & editing, Writing – original draft, Validation. **Ryan DiGiovanni:** Writing – review & editing, Supervision. **Peter Hsiue:** Writing – review & editing, Supervision, Project administration, Methodology, Conceptualization.

## Conflict of interest

M.S. Kim has stock or stock options in Restor3d. R. DiGiovanni is a paid consultant for ENOVIS; all other authors declare no potential conflicts of interest.

For full disclosure statements refer to https://doi.org/10.1016/j.artd.2026.102064.

## Figures and Tables

**Appendix 1:** ICD-10-CM codes for complications used in propensity score matching

Diagnosis	Codes
Acute myocardial infarction	I21
Heart failure	I50
Peripheral vascular disease	I73.9
Dementia	F03
Chronic obstructive pulmonary disease	J44.9
Diabetes mellitus	E08-13
Peptic ulcer	K27
Cancer	C80.1, C79
Human immunodeficiency virus disease	B20
Tobacco use	Z72.0
Liver disease	K76.9
Chronic kidney disease	N18
Cerebral infarction	I63
Rheumatoid arthritis	M06.9

ICD-10-CM, International Classification of Diseases, 10th Revision, Clinical Modification.

## Appendix A Supplementary data

Conflict of Interest Statement for Brunette

Conflict of Interest Statement for DiGiovanni

Conflict of Interest Statement for Hsiue

Conflict of Interest Statement for Kim

Conflict of Interest Statement for Pham

Conflict of Interest Statement for Soriano
