# Supplementary material for: Trends and Outcomes in Technology-Assisted Total Knee Arthroplasty in the United States From 2020 to 2024
Source: Arthroplast Today. 2026 Jun 19;40:102064. doi: 10.1016/j.artd.2026.102064 (PMC13312537; doi:10.1016/j.artd.2026.102064)
Supplement: Conflict of Interest Statement for Brunette [file mmc1.pdf]

INDIVIDUAL CONFLICT OF INTEREST STATEMENT

American Association of Hip and Knee Surgeons

(Adopted from the American Academy of Orthopaedic Surgeons disclosure statement)

The following form **must be filled out completely and submitted by each author (example, 6 authors, 6 forms). All items require a response. If there is no relevant disclosure for a given item, enter "*None*."**

Manuscript Title Trends and Outcomes in Technology-Assisted Total Knee Arthroplasty in the United States from 2020-2024

1. Royalties from a company or supplier (The following conflicts were disclosed)

   n/a

2. Speakers bureau/paid presentations for a company or supplier (The following conflicts were disclosed)

   n/a

3A. Paid employee for a company or supplier (The following conflicts were disclosed)

   n/a

3B. Paid consultant for a company or supplier (The following conflicts were disclosed)

   n/a

3C. Unpaid consultants for a company or supplier (The following conflicts were disclosed)

   n/a

4. Stock or stock options in a company or supplier (The following conflicts were disclosed)

   n/a

5. Research support from a company or supplier as a Principal Investigator (The following conflicts were disclosed)

   n/a

6. Other financial or material support from a company or supplier (The following conflicts were disclosed)

   n/a

7. Royalties, financial or material support from publishers (The following conflicts were disclosed)

   n/a

8. Medical/Orthopaedic publications editorial/governing board (The following conflicts were disclosed)

   n/a

9. Board member/committee appointments for a society (The following conflicts were disclosed)

   n/a

Each author must sign AND print or type his/her name, date and submit a separate form

In addition, one BLINDED Conflict of Interest form (no author names used) should be submitted per manuscript with all author disclosures.

Madison Brunette [signature] 10/7/25

Author Name (Print or Type)	Author Signature	Date
